# The dilemma of coordinated communication in China’s e-cigarette governance: A computational discourse analysis of a social media controversy

**DOI:** 10.18332/tid/215389

**Published:** 2026-02-14

**Authors:** Zhangyan Li, Yanhe Zhao, Xingrui Wang, Lingzhe Gao, Xingye Yao, Yu Chen

**Affiliations:** 1 College of Media and International Culture, Zhejiang University, Hangzhou, China; 2 Faculty of International Media, Communication University of China, Beijing, China; 3 School of Journalism, Communication University of China, Beijing, China; 4 Bytedance Ltd., Beijing, China; 5 Fujian Minguang Software Co., Ltd., Sanming, China; 6 School of Art and Communication, Fujian Polytechnic Normal University, Fuzhou, China

**Keywords:** e-cigarette governance, coordinated communication, social media, computational discourse analysis, public health communication

## Abstract

**INTRODUCTION:**

Amid increasingly stringent e-cigarette regulations in China – including taxation, flavor bans, and advertising restrictions – coordinated communication has emerged as a key challenge in tobacco control. This study investigates a viral incident involving Blackpink’s Jennie to explore how failures in strategic narrative coordination have undermined the legitimacy of regulatory efforts.

**METHODS:**

This study combined web scraping, large language models (LLMs) topic modeling, and critical discourse analysis (CDA) to collect and analyze an e-cigarette-related event on the Chinese social media platform Weibo from July to October 2024.

**RESULTS:**

Findings reveal that although e-cigarette-related content was widely circulated, public discourse largely lacked critical health framing. Instead, discussions often shifted toward moral judgments, cultural identity, and individual freedoms. This discursive vacuum weakened the normative foundation of tobacco control and enabled counter-narratives that questioned the state’s regulatory intent.

**CONCLUSIONS:**

The study introduces ‘coordinated communication’ as both an analytical framework and a practical imperative for effective tobacco governance. Our Research argues that legal regulation must be accompanied by proactive narrative leadership to sustain public health legitimacy. A multi-stakeholder governance mechanism involving health authorities, media institutions, and digital platforms is recommended to rebuild a coherent, health-centered public discourse in the digital

## INTRODUCTION

Since electronic nicotine delivery systems (ENDS) entered the global market in the mid-2000s, tobacco consumption has significantly shifted toward alternatives, especially e-cigarettes. Defined as battery-powered devices delivering nicotine vapor without burning tobacco, e-cigarettes have rapidly gained popularity worldwide. In China – both a major producer and exporter of e-cigarettes – the domestic market has experienced exponential growth, with the number of users reaching 16.9 million by 2023^1^.

Despite China’s efforts to tighten regulatory frameworks around e-cigarettes, the effectiveness of content control in digital spaces remains highly contested. Prior studies suggest that promotional content for e-cigarettes continues to circulate in ways that circumvent regulatory scrutiny. Chu et al.^2^, Kong et al.^3^, and Alpert et al.^4^ have demonstrated how e-cigarette companies leverage commercial websites, blogs, video platforms, and social media – such as Weibo – to disseminate advertisements. Yet this alone does not fully account for the apparent weakness of e-cigarette governance in China, a country with demonstrated capabilities in information management, as seen in other domains such as narcotics control.

This paradox raises a critical research question: ‘Why does discourse governance on e-cigarettes appear so limited in a state known for its centralized control over public opinion?’. From a strategic standpoint, the Chinese government has long framed tobacco control as a key component of its national public health agenda. Since the Reform and Opening-up period, and particularly in recent years, regulatory efforts have intensified: by 2023, 44 cities had passed or revised municipal smoking regulations; by 2024, 24 provinces and 254 cities had established local tobacco control laws. However, such institutional efforts have not translated into discursive authority over e-cigarette narratives in digital media^4^.

### Conceptualizing discursive power and coordination mechanisms in Chinese social media

Previous scholars^5^, have found that the Chinese government’s information management mechanism is highly sophisticated, possessing both top-down and bottom-up mechanisms to construct discursive consensus^6^. However, in terms of e-cigarette governance, there is currently no research showing that the Chinese government is actively working to build a unified tobacco control consensus. Drawing on the ‘coordinated communication’ framework proposed by Wei and Li^7^, our research reveals a significant absence of government discursive intervention.

The concept of Coordinated Communication proposed by Wei and Li^7^ offers a novel theoretical framework for analyzing the construction and operation of discursive power within China’s media system. Although the framework was initially developed to examine China’s international communication strategies, the authors also point out that it can be effectively applied to analyze the internal discursive power structures of a country. The theory emphasizes the role of multi-actor collaboration, systemic operation, and coordinated efficiency in shaping communication outcomes. In contrast to traditional linear models of communication, coordinated communication highlights the diversity and interactivity of communicative actors and is characterized by three core features: multi-party collaboration, systemic integration, and overall effectiveness. Since this theory was proposed at the end of 2024, it has attracted widespread attention in the Chinese communication community and has been listed as one of the important Chinese communication theories proposed in 2024^8^.

According to Wei and Li, coordinated communication refers to the collaborative participation of various actors – including government institutions, mainstream media, enterprises, civil society organizations, and the public – in the process of information dissemination. This model emphasizes that in the complex digital space, the role of mainstream media alone is insufficient. Instead, the value of other actors should also be fully considered, enabling functional differentiation and resource integration, so that national discursive power can form an efficient communication network. This would enhance the strength and persuasiveness of state discourse.

Actor coordination focuses on the dynamic interactions among different participants in the communication process. Within the framework of communication ecology, each actor plays a unique yet complementary role, and the stability of the system depends on their strategic coordination and integrative capacity^9^. Effective actor coordination requires not only the participation of multiple stakeholders but also the formation of operational synergy in content production^10^.

Content coordination comprises three layers: issue coordination, framing coordination, and rhetorical coordination. While rhetorical coordination focuses on micro-level stylistic strategies, the current study concentrates on issue and framing coordination, both of which relate directly to the distribution of discursive power.

Issue coordination involves the strategic integration of multiple related topics within the communication process to enhance agenda-setting impact. Grounded in agenda-setting theory, this concept builds upon classical models such as the issue-attention cycle, public arena model, and zero-sum theory – all of which highlight the competitive dynamics of issue salience^11^. In contrast, issue coordination proposes that issues may also function synergistically, not merely in competition^12^. It advocates for the careful planning of issue interrelationships and sequencing to optimize communicative impact.

Framing coordination refers to the alignment of narrative perspectives and interpretive schemas across actors and platforms^13^. While agenda-setting theory addresses what issues are covered, framing theory focuses on how they are presented – how narrative choices influence audience perceptions, cognitive frameworks, and emotional responses. This second-level agenda-setting function is critical in shaping public understanding and policy reception.

Together, these dimensions form the idealized model of coordinated communication, which assumes a structurally coherent discursive regime where multiple actors adopt consistent, complementary issue and framing strategies to attract audiences and convey state-sanctioned values. However, empirical studies suggest that this coordination is far from seamless.

As Chen and Gao^14^ have pointed out, ‘public discourse in China is far from monolithic’, and the state’s discursive control is constantly challenged by evolving forms of public response and creative expression. Even in ostensibly regulated online spaces, users demonstrate considerable agency and interactional flexibility. Schneider^15^ similarly observes that audiences ‘can and do reject dominant narratives, as they so often do’.

Thus, the effectiveness of coordinated communication – and the corresponding construction of discursive power – depends not only on the government’s willingness to lead, but also on the responsive alignment of mainstream media and the degree of public receptivity and affective resonance with the promoted discourses. From this perspective, the current study seeks to assess the structural realities of discursive coordination in China by asking: ‘To what extent is the Chinese government capable of mobilizing diverse communicative actors to collaboratively define the issues and frames surrounding e-cigarettes in ways that are consistent with its broader tobacco control policies?’.

## METHODS

This study combined web scraping, large language models (LLMs) topic modeling, and critical discourse analysis (CDA) to collect and analyze an e-cigarette-related event on the Chinese social media platform Weibo from July to October 2024.

### Case selection

To investigate the mechanisms and effectiveness of coordinated communication, this study selected a high-profile public opinion incident that generated widespread user engagement on Chinese social media. On 9 July 2024, media outlets reported that Jennie, a prominent member of the South Korean girl group Blackpink, was allegedly seen vaping indoors. The news quickly went viral, sparking heated debate on the Chinese microblogging platform Weibo, where it rapidly climbed to the top of the trending topics list. Jennie has 3.92 million followers on Weibo, and discussions surrounding her smoking behavior have generated several high-traffic topics on the platform. Notably, the hashtags #Jennie’s Team Admits Smoking And Apologizes and #Jennie Allegedly Smoking have together amassed a total of 510 million views, 81000 discussions, and 529000 interactions in four months. This incident provided a rare opportunity to observe discursive practices related to e-cigarettes, as a diverse range of communicative actors engaged in visible exchanges of opinion on a large public platform.

### Data collection

This research employs a multi-step method to capture the complex discursive strategies surrounding e-cigarettes in Chinese digital spaces. First, by integrating web-scraping techniques, clustering algorithms, and large language models (LLMs), we systematically collected and analyzed user-generated comments from real-time online environments. We utilized the Qingtian Multilingual Analysis Platform, using the keyword ‘Jennie e-cigarette’ to retrieve relevant content. A total of 7014 posts were collected between 8 July 2024 (the day the incident gained public attention) and 1 October 2024, originating from 17 media outlets. Among these, Weibo accounted for the majority, contributing 6240 posts (89% of the dataset). As a result, Weibo was selected as the primary platform for further analysis. The Qingtian platform also categorizes media outlets into central (state-owned) and provincial (regional) levels, enabling us to distinguish between different types of institutional sources in the subsequent analysis.

### Topic recognition

Building on these extracted elements, we further employed clustering algorithms and community detection methods to systematically identify cohesive thematic clusters within the discourse. Specifically, we first used LLMs to generate semantic tags for each user comment, capturing nuanced thematic elements. These LLM-generated tags formed the foundational nodes of our co-occurrence network, where edges represented the frequency with which two tags appeared together across the dataset.

Using the Louvain algorithm for community detection, we identified densely connected subgroups of these tags, each corresponding to a distinct discursive frame. This network-based clustering approach complemented the LLM’s tag generation by revealing latent semantic associations – groups of tags that frequently co-occurred, even if not explicitly linked in surface-level descriptions.

Clusters were validated through two means: 1) by calculating the average semantic similarity of tags within each cluster using BERT embeddings (retaining clusters with similarity scores >0.75), and 2) by examining the network’s modularity to ensure statistically significant community structures (modularity >0.4). These steps ensured that the identified discursive strategies, such as ‘diversion to systemic issues’ or ‘cultural difference justification’, were both semantically coherent and structurally distinct in the network.

Once these core discursive strategies were defined, the LLM was fine-tuned on manually labeled examples of each strategy to enable supervised classification, allowing us to map their distribution across different communicative actors (e.g. official media, netizens, fan groups) and temporal phases of the discussion. This integration of LLM-generated semantic tagging, unsupervised network clustering, and supervised classification strengthened the robustness of our frame identification, bridging algorithmic objectivity with contextual nuance.

We designed prompts to enable the large language model (LLM) to perform classification tasks^16^. Specifically, we employed a strategy known as prompt perturbation, which involves iterative and incremental modifications to prompts in order to enhance the model’s performance. Additionally, our approach instructed the LLM to adopt a researcher persona, a technique demonstrated to improve task accuracy in prior studies^17^. Subsequently, we instructed the LLM to identify latent frames within the collected texts. Previous studies have shown that, compared to baseline methods, LLM-based approaches yield more accurate and interpretable results in frame identification tasks^18^. Once key frames were extracted, we grouped semantically similar frames into overarching issues and conducted co-occurrence analysis to examine the relationships among them. We then used the LLM to classify each individual post into its corresponding frame, which allowed us to calculate the frequency distribution of each framing strategy. We employed DeepSeek-V3 as the core annotation model, given its notable effectiveness in Chinese-language classification scenarios. With a scale of 671B parameters, DeepSeek-V3 exhibits strong generalization and reasoning abilities. The model surpassed GPT-4 in binary sentiment classification (achieving 99% compared to 87.9%) and demonstrated higher accuracy (81.3%) and recall in topic classification, reflecting its strength in retrieving relevant examples across categories^19^.

### Critical discourse analysis

Finally, we conducted a critical discourse analysis (CDA) informed by the coordinated communication framework proposed by Wei and Li^7^, focusing on the qualitative examination of discursive power dynamics. Our analysis centered on the tension between mainstream media narratives and grassroots public discourse, with a particular emphasis on whether any coordination effects could be observed. This enabled us to evaluate whether the Chinese government, as both a policy promoter and a communicative actor, effectively aligned public sentiment on the issue of e-cigarettes, or whether it encountered substantial discursive resistance from the public.

## RESULTS

### Actor coordination: The absence of mainstream media and the neutrality of narrative content

Using a leading Chinese media search platform, we conducted a systematic retrieval of all reports issued by government-affiliated media outlets concerning the incident. Among central-level media, only People’s Daily published a report on 21 September 2024, more than two months after the event. Other reports came primarily from provincial or local outlets such as Minnan Net and Sichuan Online. The overall delayed response from national-level media revealed a strikingly low level of engagement during the initial phase of public discourse, which directly contradicts the theoretical expectation that mainstream media should be proactive policy communicators within a coordinated system.

Further discourse analysis shows that these reports generally adopted a neutral tone and failed to reflect the government’s tobacco control stance. For instance, the People’s Daily article began by recounting the origins of the incident and then extensively quoted Jennie’s interview in Harper’s BAZAAR (US edition), wherein she attributed her use of e-cigarettes to ‘cultural and historical differences’. The article concluded by summarizing the spectrum of online public opinion, presenting supportive and critical voices with equal weight, without offering any explicit evaluative stance on the incident or articulating a clear policy perspective.

Similar narrative patterns were observed in local media reports, which predominantly focused on the fragmentation of online opinion rather than offering guidance from a public health policy standpoint. This tendency to present sentiment rather than transmit policy suggests a broader deviation from the communicative role assigned to mainstream media under coordinated communication theory. Rather than mobilizing public discourse in alignment with tobacco control goals, these reports primarily served a ‘sentiment translation’ function, not a ‘policy mobilization’ function.

And then, our investigation into the messaging practices of official government social media accounts, revealed an even more perplexing picture. While several posts used the trending hashtag ‘#Jennie suspected of smoking#’, the actual content of these posts bore no relation to e-cigarettes. Instead, they discussed unrelated topics such as how menopausal women can manage negative emotions, why tuberculosis patients should seek standard treatment, and the benefits of foot baths in summer for those with digestive issues. This indicates not merely a lack of coordination but a total disconnection from the issue at hand.

Additionally, we found that other potential communicative actors including corporations, universities, websites, civic organizations, and app platforms, remained entirely silent on the issue. None of these entities issued any commentary or engaged with the public debate. These findings clearly demonstrate that, in the context of this e-cigarette controversy, China’s media system exhibited no signs of coordinated intent or mobilized response. Consequently, the discursive space was ceded entirely to public voices, indicating a fundamental absence of state-led narrative control in this instance.

Content coordination: Polarization and defocused public discourse undermine the harms of e-cigarettes

Given the absence of mainstream media coordination, our subsequent analysis centers on the degree of content coordination present within public discourse on social media. By employing topic modeling and qualitative coding, we identified a fragmented landscape of narrative strategies. These strategies were categorized into three overarching types: criticism, defense, and diversion, each encompassing a set of rhetorical patterns aimed at either reinforcing or diluting the moral framing of the incident (Table 1).

**Table 1:** Agenda and framing modeling

*Agenda setting*	*Framing*	*Description*
**Critical strategies**	Normative criticism	Criticizing the celebrity for setting a poor example, public figures are expected to act responsibly.
	Legal accusation	Questioning whether the behavior violates indoor smoking laws or public health regulations.
	Politeness violation	Claiming that exhaling smoke toward staff is disrespectful.
	Generalized industry critique	Using the incident to criticize the broader entertainment industry, asserting such behavior is systemic.
	Fan community accusation	Blaming fans for enabling harmful behavior through blind support.
**Defensive strategies**	Privacy defense	Framing the act as part of the celebrity’s private life, not subject to public scrutiny.
	Normalization	Downplaying the incident as a common, everyday behavior (e.g. ‘Adults smoke, it’s normal’).
	Cultural relativism	Citing cultural differences (e.g. overseas artists may have different habits or norms).
	Occupational stress defense	Justifying the act as stress relief due to the pressures of being a celebrity.
	Misunderstanding emphasis	Claiming the act was a misunderstanding, e.g. smoking while searching for lip products.
	Minimization of harm	Asserting that the makeup artist was also a smoker and didn’t mind, implying the backlash is exaggerated.
**Diversionary strategies**	Work-centric redirection	Redirecting attention to the artist’s professional work or achievements (e.g. ‘Let the work speak’).
	Comparative mitigation	Comparing the incident to worse behaviors by others to minimize the perceived severity (e.g. ‘X did something more outrageous’).

The qualitative analysis of Weibo discourse revealed three dominant types of responses to the celebrity e-cigarette incident: disciplinary, defensive, and diversionary discursive strategies. Each strategy exhibited internal variations and specific rhetorical patterns.

The disciplinary discourse primarily reflected public alignment with mainstream values and legal norms. A notable pattern was the invocation of social norms and moral expectations, wherein users emphasized that celebrities, as public figures, must lead by example and abide by mainstream codes of conduct. For instance, a widely circulated comment stated: ‘Leading minors astray – most of her fans are probably underage’. Another common strategy involved explicit legal accusations, pointing directly to violations of specific regulations. One post read: ‘This isn’t about whether she smokes, it’s about smoking indoors being prohibited, and she smoked indoors’.

In addition, some discourse emphasized violations of social etiquette, particularly focusing on the act of blowing smoke at staff members. As one user wrote: ‘Does she not see the staff as human? I feel bad for them’, and another stated: ‘Smoking is fine – it’s a personal freedom – but smoking indoors and blowing it at someone is just too much’.

Beyond specific behavioral critiques, many posts adopted a generalized critical stance, extending the critique from the individual incident to the broader entertainment industry. This included structural moral denunciations of celebrity culture and institutional practices. In some cases, users targeted fan communities, accusing them of enabling or defending inappropriate behavior: ‘Stop trying to whitewash this. I didn’t think it was a big deal at first, but the fans arguing back makes it worse’.

In contrast, the defensive discourse displayed what might be termed a tactical resistance to dominant disciplinary narratives. One prominent defense involved appeals to privacy, positioning the event as part of a celebrity’s private life and critiquing media intrusion. As one post stated: ‘Even private matters are known by the media now; celebrities really have no privacy left’.

Another common defense involved normalizing the behavior by portraying smoking as a regular, everyday action: ‘Adults smoking is personal freedom. This excessive criticism is a witch hunt’. In some posts, cultural difference was cited to contextualize the act: ‘Korean celebrities might not be used to the local smoking restrictions’. Others invoked occupational stress, suggesting the behavior was understandable given industry pressures: ‘Celebrities are under a lot of stress. Smoking to release pressure is human nature’.

Some posts attempted to reinterpret the event details to reduce moral blame: ‘It was a special situation – her makeup artist was looking for a product at the time’. A number of defenses also tried to downplay harm by emphasizing staff consent or the celebrity’s apology: ‘It was in a private space, and she asked the staff first – they said it was okay’, or ‘She already apologized. Don’t keep holding onto it’.

Finally, diversionary discourse aimed to tactically shift attention away from the controversy. One approach involved highlighting the celebrity’s professional accomplishments, often flooding the topic with positive records of artistic output, thereby attempting to neutralize the negative narrative. Another strategy employed comparative framing, particularly with regard to gender dynamics. Posts such as ‘Don’t be so harsh on female artists’ and ‘A female idol gets attacked for smoking, but male celebrities break the law and still get coddled’, repositioned the incident within broader discussions of gender bias and double standards.

These three discursive formations: disciplinary, defensive, and diversionary, constituted distinct yet interrelated modes of engagement with the incident. Each drew on specific rhetorical resources: moral norms, legal reasoning, personal emotion, or structural critique, to shape meaning and influence public reception. This discursive polarization and thematic defocusing contributed to the weakening of the public’s awareness of the actual health risks associated with e-cigarettes. Instead of reinforcing the public health logic embedded in China’s tobacco control policy, the discourse became a site of moral relativism and entertainment-driven debate. The lack of consistent framing across actors and narratives indicates a failure of content coordination, in sharp contrast to the cohesive messaging envisioned in coordinated communication theory.

**Figure 1:**
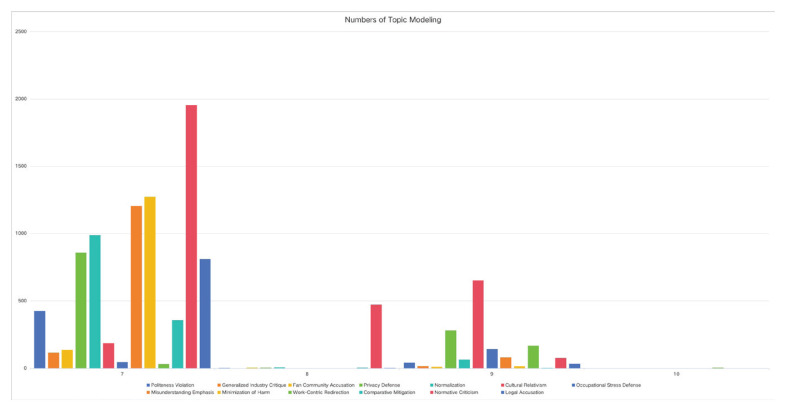


**Figure 2:**
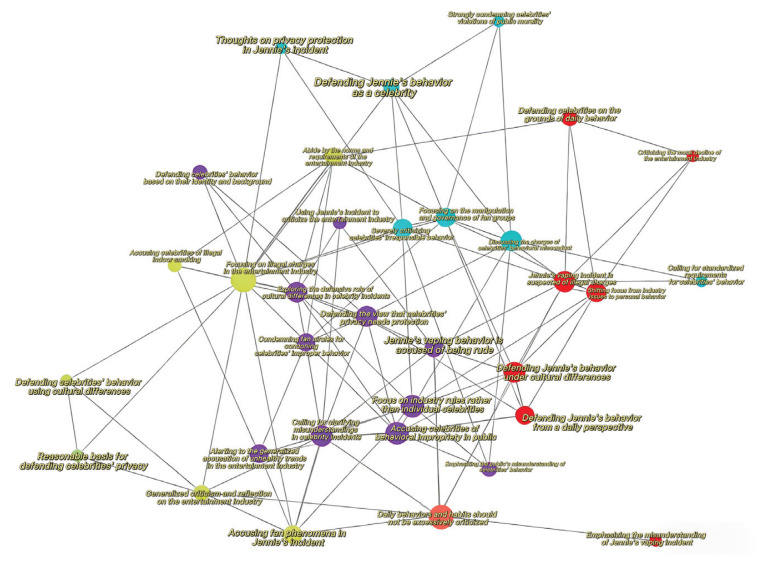


The temporal dynamics of different discursive strategies exhibit a high degree of similarity with the trends observed in the distribution of actors involved. Among all strategies, Generalized Industry Critique received the highest number of comments, followed by Minimization of Harm and Misunderstanding Emphasis. This suggests that both critical and defensive strategies possess substantial discursive power in the online debate. Rather than operating in isolation, these strategies are deeply entangled, forming a complex field of discursive confrontation (Figures 1 and 2; and Supplementary file Table 1).

Our co-occurrence frequency analysis further substantiates the relationship between different issues and frames. The findings indicate a high level of co-occurrence between defensive and diversionary discourse patterns, suggesting that these narrative strategies have converged to form a unified front against critical discourse. This convergence reveals that, in contrast to the ambiguity observed in mainstream media coverage, discourse on social media is characterized by polarization, rather than fair and balanced deliberation (Figure 2).

Such polarization runs counter to the foundational logic of coordinated communication, which emphasizes integration, complementarity, and discursive synergy. Instead, polarized discourse often entails heightened conflict and antagonism, leading to an increase in incivility within public exchanges. As Gervais^20^ notes, exposure to uncivil discourse – especially in emotionally charged debates – can normalize such behavior for portions of the audience, thereby increasing the overall tolerance for incivility in public discourse. This contributes to a more fragmented and confrontational communicative environment, further undermining the possibility of discursive coordination or policy consensus.

Our co-occurrence frequency analysis yielded similar conclusions to Gervais^20^. In the absence of proactive agenda-setting by the government, public discourse exhibited a clear pattern of polarization. Discourse strategies of the same type tended to appear together frequently – for instance, defensive strategies (marked in red) and critical strategies (marked in purple) both showed high internal co-occurrence, suggesting the formation of internally consistent discursive camps.

Moreover, these discursive camps often stood in direct opposition to one another, forming patterns of mutual rebuttal. This dynamic reinforces the polarized nature of the discourse landscape, where arguments are not negotiated or synthesized, but rather positioned in antagonistic relation. The overall structure suggests a fragmented public sphere in which discourse alignment occurs within clusters, but coordination across differing positions is absent.

The qualitative analysis of Weibo discourse during the e-cigarette controversy identified a lack of observable coordination between state-led tobacco control messaging and grassroots-level public narratives.

Public discussions on Weibo widely normalized the act of vaping. E-cigarette use was frequently framed as a matter of ‘personal behavior’ or ‘lifestyle choice’, rather than as a public health issue. References to ‘individual freedom’ were common, with this rhetorical structure often taking precedence over narratives concerning regulation or health risk.

Mentions of health-related concerns were largely absent or marginalized within the examined posts. Instead, two main types of frames – defensive and diversionary –were identified, both of which shared a common discursive strategy: the avoidance of direct engagement with the health risks associated with e-cigarettes. These frames often redirected attention toward the subjectivity or identity of the user. Discussions centered on moral evaluations such as whether vaping constituted improper behavior, poor etiquette, or disrespect toward others.

Within diversionary frames in particular, the act of vaping was made less visible. The focus shifted toward the emotional states of individuals or the moral standards of celebrities, rather than the act of vaping itself. This discursive redirection corresponded with a structural shift in the issue’s framing. Three major discursive shifts were observed in grassroots narratives:

1. De-politicization – the detachment of vaping discourse from policy contexts;
2. De-medicalization – the marginalization of health-related concerns; and
3. Re-moralization – the redefinition of the issue through moral and behavioral lenses.

These patterns illustrate a consistent discursive transformation in which grassroots users reframed e-cigarette discussions away from public health and toward individualistic or symbolic domains.

## DISCUSSION

This study reveals a phenomenon of critical practical significance: despite the Chinese government’s increasingly stringent regulatory measures on e-cigarettes in recent years, it has failed to establish a correspondingly robust discursive mechanism for public guidance and cognitive intervention. This policy–discourse disjunction manifests as a growing gap between formal regulatory rigor and the discursive weakness of state-affiliated communicators. On one hand, policy documents and legal frameworks have become increasingly comprehensive and restrictive; on the other hand, mainstream media and official dissemination channels have shown minimal engagement with the e-cigarette issue. Their discourse remains largely neutral, depoliticized, and low in agency, effectively ceding narrative control to grassroots voices.

The immediate consequence of this discursive vacuum is that public understanding of e-cigarettes is largely shaped by fragmented online discussions and anecdotal interpretations rather than a coherent framework of health knowledge. The absence of a state-led discursive infrastructure has not only weakened the public legitimacy of anti-smoking policies but also contributed to a semantic drift, where the issue of tobacco control loses its footing as a public health concern. In many discussions, the media’s occasional coverage of e-cigarettes is dismissed by users as ‘unnecessary meddling’ or ‘excessive paternalism’, with calls to relax tobacco restrictions in the name of adult autonomy and personal freedom. This reframing marks a significant shift: from the empirical harms of e-cigarettes as a factual concern to abstract debates over freedom and individual rights.

From the perspective of agenda-setting theory, this shift signifies a diversion from substantive health concerns toward broader critiques of governmental authority and media credibility. Public discourse has moved from asking: ‘Are e-cigarettes harmful?’ to ‘Should the government intervene?’ and ‘Is this an infringement on adult choice?’– a discursive redirection that undermines both policy clarity and public health legitimacy.

In light of these findings, we advocate for a more proactive exercise of discursive power by the Chinese government. Specifically, public health authorities should release timely, accessible information highlighting the health risks of e-cigarettes during moments of public attention, while mainstream media should be mobilized to echo and amplify these messages. Such coordination could restore the agenda-setting function of state-affiliated media and offer support to grassroots voices already opposing e-cigarettes, thereby anchoring public discourse within a health-oriented frame.

However, we express a deeper concern that this failure of coordination may not merely reflect a lack of capacity, but rather a lack of political will. As earlier studies have noted, entrenched institutional entanglements between regulatory authorities and the tobacco industry – particularly the dual identity of China’s State Tobacco Monopoly Administration (STMA) as both regulator and stakeholder – continue to shape the policy environment. In this context, the government’s silence or neutrality on e-cigarettes may represent a deliberate form of discursive abstention. Out of concern for industrial interests, public sentiment, and political risk, the state may be strategically refraining from framing e-cigarettes as a public health crisis, thus relinquishing its narrative leadership. This may also explain why covert marketing of e-cigarettes continues on digital platforms with little evidence of systematic takedowns or algorithmic suppression^21^. Therefore, echoing prior scholarship, we call for the establishment of a multi-actor collaborative governance mechanism – one that incorporates public health authorities, mainstream media, social media platforms, and civil society stakeholders. Such a mechanism could enhance the media system’s sensitivity and professionalism on tobacco-related topics, promoting a discursive framework grounded in public health rather than vested interests.

### Strengths and limitations

This study offers several notable strengths across theoretical, methodological, and practical dimensions. Theoretical strength lies in its conceptualization and operationalization of ‘Coordinated Communication’ within the context of discursive power construction in China. By situating this emerging concept in the Chinese media system, the study establishes a novel analytical lens applicable not only to public health but also to other issue areas within highly centralized communication environments. This framework enhances scholarly understanding of the dynamic interactions among state, media, and the public in agenda-setting processes.

Methodologically, the study develops an innovative multi-step method design that combines computational communication techniques with critical discourse analysis (CDA). Through topic modeling and large language model-assisted classification, it identifies key topics and dominant frames in online discussions. CDA is then used to interpret underlying tensions and power structures within these frames. This approach is particularly suited for exploring contested narratives in complex or polarized issue domains. Practically, the study highlights a critical yet understudied gap in China’s tobacco control efforts: the absence of coordinated discursive intervention by mainstream media. Despite increasingly strict regulations, insufficient media engagement has allowed public narratives to drift away from health-centered framing, weakening policy influence. The study underscores the importance of discursive legitimacy in policy implementation and offers actionable recommendations such as enhancing narrative leadership by public health authorities and news media, to support more effective communication strategies in tobacco regulation.

This study has certain limitations. First, as a case study, it cannot fully represent the extent of the Chinese government’s engagement with e-cigarette discourse. Rather, it offers an analytical perspective that calls for further research drawing on a broader range of cases to assess the government’s efforts and to develop a more robust evaluation framework. Second, as a commercial social media platform, Weibo’s discussions may be influenced by financial incentives. For example, BlackPink’s management company and fan communities could potentially intervene in the discourse, steering it toward a more favorable tone or diverting attention from the core issue. This is inherently a multi-stakeholder bargaining process. Our study focuses only on surface-level descriptions and lacks a deeper examination of the underlying dynamics, which warrants further investigation.

## CONCLUSIONS

This study identifies the discursive synergy present in public discussions surrounding e-cigarettes in China. Through computational social science analysis, we found that mainstream Chinese media exhibited limited responsiveness to e-cigarette-related emergencies, while public discourse tended to drift away from the issue’s public health dimensions, showing relatively low concern for smoking behaviors. We recommend that mainstream media play a more active role in shaping the public agenda on e-cigarettes, thereby contributing to more effective media-driven governance in China’s e-cigarette regulation efforts.

## Supplementary Material



## Data Availability

The data supporting this research are available from the following sources: https://github.com/w8692736/Jennie-Electronic-Cigarette-Public-Opinion-Analysis-System
