# Supplementary material for: The dilemma of coordinated communication in China’s e-cigarette governance: A computational discourse analysis of a social media controversy
Source: Tob Induc Dis. 2026 Feb 14;24:10.18332/tid/215389. doi: 10.18332/tid/215389 (PMC12906255; doi:10.18332/tid/215389)
Supplement: Supplementary file 1 [file TID-24-20-s1.pdf]

Supplementary file

Table 1: Theme change over time

	week_1	week_2	week_3	week_4	week_5	week_6	week_7	week_8	week_9	week_10	week_11	week_12	week_13	0
Normative Criticism	1921	27	4	6	433	3	34	2	1	0	64	13	0	2508
Legal Accusation	786	21	2	4	1	1	0	1	1	0	29	3	0	849
Politeness Violation	402	19	2	4	1	1	0	0	0	0	40	2	0	471
Generalized Industry Critique	108	6	2	0	0	0	0	0	0	0	16	0	0	132
Fan Community Accusation	133	4	0	0	0	0	6	0	0	0	6	4	0	153

	week_1	week_2	week_3	week_4	week_5	week_6	week_7	week_8	week_9	week_10	week_11	week_12	week_13	SUM
Privacy Defense	850	7	3	0	0	0	3	2	1	1	271	8	0	1146
Normalization	979	7	2	0	0	1	0	2	0	2	60	3	0	1056
Cultural Relativism	185	2	1	0	0	0	0	0	0	2	635	15	0	840
Occupational Stress Defense	46	1	0	0	0	0	0	0	0	0	139	5	0	191
Misunderstanding Emphasis	1187	17	1	1	0	0	0	0	0	0	80	1	0	1287
Minimization of Harm	1255	17	2	0	0	0	0	0	0	0	15	0	0	1289

	week_1	week_2	week_3	week_4	week_5	week_6	week_7	week_8	week_9	week_10	week_11	week_12	week_13	SUM
Work-Centric Redirection	31	0	0	0	0	0	0	0	0	0	163	5	1	200

Comparative Mitigation	345	13	1	0	0	0	1	0	0	0	4	0	0	364

© 2026 Li Z. et al.
